# Understanding barriers to well-child visit attendance among racial and ethnic minority parents

**DOI:** 10.1186/s12875-024-02442-0

**Published:** 2024-06-03

**Authors:** Nisha Fahey, Allison Holt, Deniz Cataltepe, Annelise Brochier, Amy Stern, Morgan Mazanec, James W. Courtemanche, Tracey Wilkie, Kellie Tan, Rulan Lyu, Eric Alper, Josephine Fowler, Lawrence Rhein, Arvin Garg

**Affiliations:** 1 UMass Chan Medical School, 55 Lake Ave N, Worcester, MA 01655 USA; 2 Children’s Hospital of Philadelphia, Philadelphia, PA USA; 3 Boston Medical Center, Boston, MA USA; 4 Massachusetts Health Quality Partners, Brighton, MA USA; 5 Dartmouth Health, Lebanon, NH USA; 6 UMass Memorial Health, Worcester, MA USA

**Keywords:** Well-child visits, Barriers to care, Language, Health inequity

## Abstract

**Objectives:**

To assess racial and ethnic minority parents’ perceptions about barriers to well-child visit attendance.

**Methods:**

For this cross-sectional qualitative study, we recruited parents of pediatric primary care patients who were overdue for a well-child visit from the largest safety net healthcare organization in central Massachusetts to participate in semi-structured interviews. The interviews focused on understanding potential knowledge, structural, and experiential barriers for well-child visit attendance. Interview content was inductively coded and directed content analysis was performed to identify themes.

**Results:**

Twenty-five racial and ethnic minority parents participated; 17 (68%) of whom identified Spanish as a primary language spoken at home. Nearly all participants identified the purpose, significance, and value of well-child visits. Structural barriers were most cited as challenges to attending well-child visits, including parking, transportation, language, appointment availability, and work/other competing priorities. While language emerged as a distinct barrier, it also exacerbated some of the structural barriers identified. Experiential barriers were cited less commonly than structural barriers and included interactions with office staff, racial/ethnic discrimination, appointment reminders, methods of communication, wait time, and interactions with providers.

**Conclusions:**

Racial and ethnic minority parents recognize the value of well-child visits; however, they commonly encounter structural barriers that limit access to care. Furthermore, a non-English primary language compounds the impact of these structural barriers. Understanding these barriers is important to inform health system policies to enhance access and delivery of pediatric care with a lens toward reducing racial and ethnic-based inequities.

**Supplementary Information:**

The online version contains supplementary material available at 10.1186/s12875-024-02442-0.

## Introduction

Annual well-child visits are the cornerstone of pediatric healthcare. The American Academy of Pediatrics and the American Academy of Family Physicians recommendations for well-child visits establish a standardized timeline for encounters with the medical system focused on preventive care [1, 2]. Well-child visits serve as an opportunity to promote child health and development by tracking growth and developmental milestones, monitoring chronic conditions, screening for medical and non-medical risk factors, administering vaccinations, and providing anticipatory guidance [3]. However, children frequently miss well-child visits [4–7] and racial-ethnic and primary language inequities in attendance rates are known to exist and contribute to inequitable health outcomes [8–13]. Understanding of the driving factors that result in lower well-child visit attendance specifically among children in racial and ethnic minority groups, however, remains limited [7, 14–16].

A safety net health system in central Massachusetts identified inequities in attendance to pediatric well-visits with Black and Latinx groups having 61.5% attendance rates compared to 71.1% rates among their White and non-Latinx counterparts. This study assessed racial and ethnic minority parents’ perceptions about well-child visits to guide the development of responsive policies to reduce inequities in this important component of pediatric healthcare.

## Methods

### Participant eligibility and recruitment

The population health team at UMass Memorial Health identified 3,186 pediatric patients across 48 UMass practice sites ages 0 – 21 years who were overdue for a well-child visit in the previous year (February 1, 2020, to January 31, 2021). Infants were considered overdue if they were projected to have fewer than six well-child visits by 15 months old. Children over 15 months of age were considered overdue if they did not have a well-child visit CPT code in any of their primary care encounters in the prior 12-month period. A recruitment letter was sent by email and/or postal mail to the family of the identified roster of patients. Additionally, recruitment flyers were posted in several of the UMass practice sites. Parents of patients who identified Spanish as a primary language and were a part of racial and ethnic minority group were purposefully recruited in order to assess the specific barriers to care that these families face given the health system’s goal to reduce racial/ethnic inequities in well-child visit attendance. We sought to interview at least 15 parents with Spanish as a primary language and a total of at least 25 participants, which is within the range of a typical sample size for qualitative studies [17]. Interested parents scheduled a telephone interview and were offered $100 upon interview completion; all parents who agreed to participate completed their interview. In this manuscript, we use “parents” to describe the parent/guardian who participated in the study.

### Development of the interview guide

A semi-structured interview guide (Appendix S1) was developed specifically for this study by a group of clinicians who care for pediatric patients as well as qualitative researchers. The guide was used to prompt discussion about pre-determined topics including overall experience with well-child visits and factors that impact access to care. Participants were asked to share their perspectives on the purpose, value, and content of well-child visits and share their barriers and facilitators for well-child visit attendance. Finally, sociodemographic information was collected verbally from the participant. The guide was composed of open-ended questions and probes that were flexible to allow the interviewer to explore issues of relevance as they emerged.

### Data collection

Twenty-five interviews were conducted by telephone between March and July 2021. The interview duration ranged from 25 to 60 min with an average of 30 min. Fifteen interviews were conducted in Spanish based on participant preference utilizing a translated interview guide and the remainder were conducted in English; all were audio-recorded, with the participant’s verbal consent. The English language interviews were conducted by a pair of Massachusetts Health Quality Partners senior researchers; one researcher served as the primary interviewer [A.S] while the second collected additional data through detailed notes [M.M]. Two bilingual interviewers [S.D. and N.K.] with backgrounds in public health who were per-diem consultants for Massachusetts Health Quality Partners performed the Spanish language interviews following the same process. All of the interviewers were female and they had no prior relationships with any of the participants. Qualitative methods, including directed content analysis, were used to explore participants’ thoughts regarding a set of previously determined interview topics. In each interview, the participant was a major speaker and the researcher served as a guide and facilitator. The semi-structured interviews yielded significantly rich data whereby no additional themes seemed to emerge, suggesting sufficient data to develop themes.

### Data transcription and analysis

Written notes taken during the interview were analyzed by three researchers using directed content analysis techniques. Inductive coding techniques were used to identify and code common themes that emerged from the interviews. These themes were quantified in order to detect the frequency with which they occurred during interviews. The frequency of emerging themes was considered an indicator of their importance. Barriers and challenges were identified as primary and secondary based on the reoccurrence of issues across study participants.

This study was deemed not human subjects research and was considered exempt by UMass Chan Medical School Institutional Review Board because data was collected anonymously and was a part of quality improvement efforts.

## Results

### Sociodemographics

Twenty-five parents of pediatric patients were interviewed with the majority (88%) being mothers. Sixty percent of the interviews were conducted in Spanish and the rest in English. Most (80%) participants identified a non-English language as one of the primary languages spoken in their home, of which 68% reported speaking Spanish. Overall, 76% of participants owned a car, 20% owned a home, and 44% were employed in some capacity. The average child's age was 8.1 years. All participants had access to the internet (Table 1).

**Table 1:** Participant sociodemographic characteristics (*N* = 25)

	n (%)
Relationship to child	
Mother	22 (88%)
Father	1 (4%)
Other	2 (8%)
Race/Ethnicity	
Black/African American	4 (16%)
Asian	3 (12%)
Hispanic or Latinx	17 (68%)
Other	1 (4%)
Primary language(s) spoken in the home	
Spanish	12 (48%)
English	5 (20%)
Spanish and English	5 (16%)
English and other language	2 (8%)
Other language	1 (4%)
Employment status	
Unemployed	14 (56%)
Full time employed	8 (32%)
Part time employed	1 (4%)
Per diem employed	1 (4%)
Student	1 (4%)
Own or lease a car	
Yes	19 (76%)
No	6 (24%)
Housing	
Own their home	5 (20%)
Rent their home	15 (60%)
Live with someone else	3 (12%)
No response	2 (8%)

### Well-child visit purpose and perceptions

Participants were asked to share their understanding of the purpose and importance of well-child visits. Participants cited an array of reasons for well-child visits and nearly every participant was able to identify the significance and value of well-visits to the overall wellbeing of their child. The most common responses regarding the purpose of these visits included monitoring overall health, “determin[ing] if any health issues exist,” monitoring the child’s growth with height and weight, “mak[ing] sure the child meets milestones,” providing preventive care, and ensuring the child is “up to date with vaccines.” Some participants pointed out that the purpose of the visit was to have a yearly physical exam; others revealed that the visit provided the opportunity to ask questions or raise concerns and have in-depth conversations about safety in the home, nutrition counseling, as well as behavioral and social health monitoring. Other aspects that were reported less frequently included monitoring and treating chronic conditions, treating an acute illness, checking vital signs, evaluating sleep patterns, following-up injuries, and receiving parenting advice.

### Primary barriers

The primary barriers to well-child visit attendance identified by parents were all structural in nature. These primary barriers include parking, transportation, language, appointment availability, and work/other competing priorities. Participants noted that “paid parking” is frequently located “far from the main entrance.” For those who do not drive to appointments, transportation often requires planning “days ahead” and can necessitate out-of-pocket payment. Furthermore, appointment availability and competing priorities also impact the accessibility of medical care. Appointments outside of school hours are booked “a year in advance” and sometimes parents have to “reschedule appointments to accommodate work.”

Language barriers present unique challenges as well as further compound the identified structural barriers. Participants noted that not having “enough staff who speak Spanish” negatively impacts their ability to utilize “valet parking,” “call the office on the phone,” and “complete forms and paperwork.” Some fear that a lack of in-person interpreters risks “important information from the doctors and nurses getting lost in translation.” Participants illustrated how these major barriers and challenges prevent them as parents from taking their children in for medical visits or consistently attending visits (Table 2).

**Table 2:** Selected parent quotations: structural barriers

Theme	Code Definition	Quotation
Parking	Any comments related to parking for appointments (i.e., availability of spots, cost of parking, distance from parking to office, safety of parking far away from building- crossing busy street, traveling in inclement weather conditions)	• “The available parking spots are a little far from the main entrance.” • “I don’t understand why there is paid parking, so I park on the street and carry the baby in the car seat down the road.” • “…there is not enough staff who speak Spanish and that there is too much traffic at the main entrance to even get to valet parking.”
Transportation	Any comments related to traveling to appointments, including public or personal transportation access (i.e., using the bus, sharing a car with others)	• “If I want my transportation covered by the insurance, I have to plan days ahead, and sometimes it doesn’t work out or sometimes the insurance loses the paperwork and I have to pay anyway.” • “We have a car, but I do not drive. When my husband works, he uses the car. When we go in a taxi, we have to go without a car seat.” • “But sometimes I have the money for a taxi and sometimes I do not. Also there is a lot of coordination needed with taking a taxi, bus, or getting a friend of the family to take us.”
Language	Any comments related to language (i.e., limited English proficiency, availability of interpreter services)	• “When I call the office on the phone there is no option to listen to the messages in Spanish or to speak to someone who speaks Spanish.” • “I am not even sure if the office has forms and paperwork available in Spanish.” • “I feel that I have received bad treatment as a Spanish speaker. The nurse takes longer, the translator won’t work. It feels they aren’t very accommodating.” • “Many people assume all Spanish is the same, and it is not, so sometimes even working with Spanish speaking interpreters is hard.” • “I’m concerned about important information from the doctors and nurses getting lost in translation. I prefer to have an in-person translator.”
Appointment availability	Any comments related to making appointments in the desired time frame and desired time of day	• “Appointments outside of school hours are booked a year in advance and only for school age kids.” • “I do not like having my child miss class.” • “There are not enough available appointments with the pediatrician.”
Work	Any comments related to disruption of the workday or work schedule to attend visit (i.e., taking time off work, calling out sick, using their lunch break)	• “…sometimes had to leave work early, sometimes had to call out sick to take the baby to the appointment.” • “I would sometimes need to reschedule appointments to accommodate work.” • “I depend on my husband for a ride. He usually moves his work schedule around so we can make it to our child’s appointments.”
Competing priorities	Any comments related to demands other than work that prevent them from attending child well-visits (i.e., childcare for other children, children attending school)	• “It’s hard for me to leave the house and care for four kids at once…Sometimes I don’t have childcare for the other children when I go to an appointment for one child.” • “I don’t like having to take my daughter out of school for doctor’s appointments.”

### Secondary barriers

Additional barriers cited less commonly that did not meet the threshold of primary barriers were experiential in nature. These barriers include interactions with office staff, racial/ethnic discrimination, appointment reminders, methods of communication, wait time, and interactions with providers. The opinions about these experiential factors varied across participants and were not consistently identified as barriers to well-child visit attendance. Some individuals expressed difficulty getting “a hold of someone” when calling the office and “not getting reminders in any form” leading them to “have to call [themselves] to schedule the well-child checks.” Those who reported receiving appointment reminders found them helpful, but expressed preferences for specific forms on communication, such as phone calls or text messages. Sometimes “long waits” at the office can be “an issue” because some families depend on “the bus” and “rides” from others for transportation. When asked about their experience of racial or ethnic discrimination, participants occasionally expressed that there is a “preconception of my needs or information that I need” based on race or ethnicity.

### Impacts of COVID-19 pandemic

Parents’ reactions to COVID-19 and the impact the pandemic had on attending well-child visits varied. In some cases, participants were fearful of in-person visits while others reported no concerns. Some participants had difficulties scheduling in-person appointments. Others opted to use telehealth instead, though use and experiences differed within the group. Overall, the COVID-19 pandemic was not consistently identified as a factor impacting well-child visit attendance, compared to the structural barriers described above, among this cohort.

## Discussion

This study demonstrates that racial and ethnic minority parents in this cohort considered well-child visits to be an important aspect of their child’s wellbeing; however, structural barriers often impeded adherence to the pediatric well-child visit schedule and need to be addressed to improve access to care and reduce health inequities. The primary barriers identified were parking, transportation, appointment availability, language, and work/other competing priorities. These barriers are largely in line with those identified in prior studies conducted at various health centers across the United States and beginning as early as the 1990s, suggesting that these obstacles have persistently effected well-child visit attendance across the country [6, 10, 18, 19]. We also found that language barriers intersect with and exacerbate several of the other structural barriers that were identified.

Our study uniquely highlights the compounding effect of language barriers on other common barriers through the voices of parents. Previous studies have identified language differences as a barrier to care; parents and caregivers reported the existence of language services to be beneficial in facilitating visit attendance [10, 16]. One study quantitatively linked parent report of culturally sensitive care with quality of well-child visits [20]. Language is a known key component of culturally sensitive care [21]. Other studies have demonstrated that language concordance between families and providers is not associated with quality of well-child visit or satisfaction with communication [22, 23]. In our study, qualitative methods illustrated the ways language barriers outside of the parent-provider interaction posed distinct challenges for parents, and also how they intersected with and exacerbated several of the other structural barriers, ultimately contributing to missed well-child visits. These parents highlighted how communication is embedded within structural barriers from scheduling appointments to valeting one’s car and significantly impacts their ability access well-child care, particularly if their primary language is not English. With this in mind, future work could take a similar approach to further explore the relationship of structural barriers impacted by language with quality of well-child visits and whether efforts to address these barriers can improve both attendance and quality of well-child visits.

It is also important to note that the interviews took place in the setting of the COVID-19 pandemic; however, the baseline data that demonstrated the existing inequity preceded the pandemic. Despite the challenges that emerged during the pandemic, it was not identified as a barrier significantly impacting well-child visit attendance. Therefore, the barriers identified likely transcend the COVID-19 pandemic.

This study is limited by sample size and single-center setting. However, the purposeful sampling of racial and ethnic minority parents, as well as the large proportion of participants who primarily speak a non-English language, are strengths and a novel addition to the current literature. It is reasonable to believe that these findings are generalizable to other clinical settings with patient populations largely composed of racial and ethnic minority groups. It is possible that those who chose to participate may value well-child visits more; however, all participants were the parent of children who had missed well-visits in the past. It is possible that these parents were influenced by a degree of social desirability in their responses to the semi-structured interview. Despite that factor, they all identified barriers based on their experience and these findings may be an underrepresentation of the negative impact of these barriers on attendance at well-child visits.

The knowledge gained by understanding the barriers experienced by racial and ethnic minority parents is of great value for health systems and pediatric practices. Critical structural barriers were identified that negatively affect access to primary care for racialized groups. Addressing these barriers may reduce the racial gap in well-child visit adherence, which also has short- and long-term implications for children’s health. For example, in response to these findings, UMass Memorial Health, has implemented multiple pilot projects to reduce these barriers. These efforts include removing parking fees for well-child visits, offering free transportation, and standardizing appointment reminders [24]. Specifically, appointment reminders were in the form of personal telephone calls at multiple time points leading up to the appointment with the assistance of a telephonic interpreter when indicated based on the patient’s preferred language in the electronic medical record [24]. Additional efforts that expand appointment availability and address competing priorities are also necessary. An overarching focus on increasing language accessibility through more bilingual staff, translated written materials, and enhanced access to interpreters can aid in overcoming a broad range of barriers identified in this study. Overall, innovative and sustainable care models are urgently needed to reduce the structural barriers that racial and ethnic minority families disproportionately face in order to achieve equity in primary care services.

### Supplementary Information

Supplementary Material 1. 

Supplementary Material 2. 

## Data Availability

All quantitative data analysed during this study are included in this published article. The qualitative data was analysed from transcripts as described in the manuscript. Full transcripts are not shared out of consideration for participant privacy.
