# Supplementary material for: Understanding barriers to well-child visit attendance among racial and ethnic minority parents
Source: BMC Prim Care. 2024 Jun 3;25:196. doi: 10.1186/s12875-024-02442-0 (PMC11149240; doi:10.1186/s12875-024-02442-0)
Supplement: Supplementary file 1 — Supplementary Material 1. [file 12875_2024_2442_MOESM1_ESM.docx]

# Appendix S1: Interview Guide

**UMASS MEMORIAL HEALTH CARE INTERVIEW GUIDE FOR BARRIERS TO ROUTINE CHILD WELL-VISITS**

*Introduction*

- Introduce interviewer and note-taker.
- MHQP is an independent, non-profit organization that brings together providers, payers and patients in Massachusetts healthcare to help improve the quality of patient care experiences throughout the state.
- MHQP is working with UMass Memorial Health Care on this project. UMass Memorial Health is interested in understanding the challenges families face getting to child well-visits. Describe the value of family input on addressing challenges to receiving care, stating that their perspectives are important in figuring out how to make it easier for families to keep their appointments.
- We are also speaking with other families.
- Review the semi-structured nature of the interview, length of interview, confidentiality, anonymity, and the elements of informed consent, including ability to skip any question/ask clarifying questions at any time, reporting and data storage/archiving
- Ask if participant would still like to participate; get verbal consent to participate.
- Request permission to audio-record.
- Any questions before we get started?
- Start recording and ask to repeat verbal permission on audio recording.

**Semi-Structured Interview**

[Introduction/Greeting]: Thank you for agreeing to speak with us. Your input is very important to us as we understand the experience of families with child well-visits. Well-visits are routine appointments with your child’s healthcare provider at set ages.

[Open-ended question to start the interview]: Can you please tell us about your experience with child well-visits?

[secondary probes if participant does not discuss]:

- What do you think is the purpose of these child well-visits?
- How important do you find these child well-visits to be?
- What are some topics that are discussed at child well-visits?

[Specific question about child well-visit attendance]

We are interested in understanding the challenges families may face with completing routine child well-visits. Can you think of a time when it was impossible or very hard to go to a child well-visit or appointment? Tell me about it. What made it so hard? What would have made it easier for you?

Probes to address barriers:

- Scheduling difficulties – availability of appointments with provider, clinic hours, scheduling staff
- Transportation
- Access to car seat
- Parking
- Child Care
- Competing priorities (e.g., not able to take time off of work)
- Financial
- Language
- COVID/Safety
- Understanding/prioritizing importance of scheduled child well-visits
- Child has been to doctor or ER when they were sick
- Long wait times to be seen at clinic

Additional follow up questions to ask (if not already discussed/addressed):

- Do you receive reminders about your upcoming well-visit appointment and/or need to schedule well-visit appointment?
- Are receiving reminders helpful? Why/why not?
- What has been your experience with the system used for reminders (e.g., phone call reminder; text reminder; email reminder)?
- Do you feel like you will be treated differently because of your race/ethnicity/gender?
- Do you feel like you will receive poorer service because of your race/ethnicity/gender?
- If you could change one thing when you bring your child for a well-visit appointment, what would it be?

*Questions about valuable data*

For this next set of questions, let’s imagine that you were going back in time and selecting where your child will be seen and who your child will see for well-visits, and you had access to any information you wanted.

- What information would you want to know about in order to make the decision? Why?
- If all information were available, would you be most interested in looking at information about a specific healthcare provider, the practice or clinic, or the hospital that the healthcare provider is affiliated with?

*Questions about demographics*

Next we would like to ask you some demographic questions. If you prefer to not answer any, please just let us know or you can say “skip”.

- Child Age (UMASS to provide?)
- What is your relationship to the child?
- How many adults currently reside in the household?
- How many children currently reside in the household?
- What is the primary language spoken at home?
- What is your employment status? [employed FT, employed PT, employed seasonally, student, not employed]
- Do you own or lease a car? If no, do you have access to a car? Is it readily available?
- Self-identified race/ethnicity
- Do you own or rent your home?
- What way(s) do you prefer to be contacted by your child’s doctor? (letter, call, email, text)
- Do you have access to the internet? Do you use the “My Chart” Patient Portal?
- Is your child currently being seen by other medical specialists (could be indication of chronic conditions)

*Other comments*

- Is there anything else you’d like us to know about your experiences with your healthcare provider?
- Do you have suggestions for how best to make it easier for families to complete their child well-visits?

*Note to interviewer: If interviewee mentions equity or racism then take note and document what they say. Probe if appropriate and ask followup questions with sensitivity.*

*Closing*

- Thank you for your input, the information you’ve shared is extremely helpful.
- Reiterate how information will be used.
- We would like to get your correct address so we can send you the stipend for participating. We will turn off the recording so this information is not captured on the audio recording.
